# Periodontal and Hepatic Parameters in Obese Patients Undergoing Bariatric Surgery

**DOI:** 10.3290/j.ohpd.b3240761

**Published:** 2022-07-22

**Authors:** Dejana Čolak, Tadeja Pintar, Alja Cmok Kučič, Jure Salobir, Boris Gašpirc, Rok Gašperšič

**Affiliations:** a PhD Student, Department of Oral Diseases and Periodontology, Dental Clinic, University Medical Centre Ljubljana, Ljubljana, Slovenia; Faculty of Medicine, University of Ljubljana, Ljubljana, Slovenia. Hypothesis, idea, study concept, data collection, statistical evaluation, wrote the manuscript.; b Professor, Department of Abdominal Surgery, University Medical Centre Ljubljana, Ljubljana, Slovenia. Performed the bariatric surgery, liver biopsies, and obtained histological analysis; hypothesis, study concept, wrote the manuscript, performed patient examination, data collection, obtained funding.; c Specialist in Periodontology, Department of Oral Diseases and Periodontology, Dental Clinic, University Medical Centre Ljubljana, Ljubljana, Slovenia; Faculty of Medicine, University of Ljubljana, Ljubljana, Slovenia. Hypothesis, study concept, data collection, contributed to writing the manuscript.; d PhD Student, Department of Abdominal Surgery, University Medical Centre Ljubljana, Ljubljana, Slovenia; Faculty of Medicine, University of Ljubljana, Ljubljana, Slovenia. Study concept, data collection, contributed to writing the manuscript.; e Professor, Department of Oral Diseases and Periodontology, Dental Clinic, University Medical Centre Ljubljana, Ljubljana, Slovenia; Faculty of Medicine, University of Ljubljana, Ljubljana, Slovenia. Idea, study concept, contributed to writing the manuscript, obtained funding..; f Professor, Department of Oral Diseases and Periodontology, Dental Clinic, University Medical Centre Ljubljana, Ljubljana, Slovenia; Faculty of Medicine, University of Ljubljana, Ljubljana, Slovenia. Performed patient examination, idea, hypothesis, study concept, wrote the manuscript, obtained funding.

**Keywords:** gingivitis, liver biopsy, metabolic associated fatty liver disease, periodontitis, risk factor

## Abstract

**Purpose:**

Current discoveries imply a connection between periodontitis and metabolic associated fatty liver disease (MAFLD). This study aimed to determine the prevalence of periodontitis and MAFLD in obese patients with BMI >40, employing the most reliable diagnostic methods, namely liver biopsy, and detailed periodontal examination.

**Materials and Methods:**

Liver biopsy and periodontal examination were performed in 30 obese patients with BMI BMI >40 undergoing bariatric surgery. Kleiner’s classification was used to determine non-alcoholic steatohepatitis (NAS) activity score, non-alcoholic steatohepatitis (NASH) and liver fibrosis. The periodontal condition was classified following the recent AAP/EFP classification. Patients were divided into periodontitis (PG) and non-periodontitis groups (NPG). Data on systemic health parameters were collected from patients’ medical records. Descriptive statistics and simple statistical tests were used to determine the differences between the two groups.

**Results:**

The prevalence of NASH in the sample was 43% (13/30), borderline NASH 37% (11/30), while fibrosis stage 1 was most common (72%, [22/30]). Periodontitis prevalence was 67% (20/30), while all non-periodontitis patients (33%; 10/30) exhibited gingivitis. PG and NPG did not differ in NAS or NASH prevalence (p > 0.05). However, the periodontitis group showed higher C-reactive protein levels, while NPG showed higher gamma-glutamyl transpeptidase levels (p < 0.05).

**Conclusion:**

The study results suggest the considerable prevalence of MAFLD, periodontitis and gingivitis in obese patients with BMI >40 undergoing bariatric surgery. Patients with periodontitis had higher CRP levels, while those with gingivitis presented higher gamma-glutamyl transpeptidase levels.

Metabolic-associated fatty liver disease (MAFLD),^17^ formerly known as non-alcoholic fatty liver disease, is a common liver condition affecting a quarter of the world’s population, with tremendous consequences on the individual’s health and life expectancy.^78^ MAFLD results from a diverse range of metabolic abnormalities and is associated with many risk factors such as obesity, metabolic syndrome, dyslipidaemia, hypertension, insulin resistance, diabetes mellitus, gut dysbiosis, in addition to age, sex, and genetic predisposition.^74,78^ In the presence of unfavourable dietary patterns, frequent variants in regulatory genes of lipid metabolism in hepatocytes, such as PNPLA3, TM6SF2, and MBOAT7, have already been linked to MAFLD.^74,79^ MAFLD progresses gradually from fatty liver (steatosis) through non-alcoholic steatohepatitis (NASH) to fibrosis, liver cirrhosis and even hepatocellular carcinoma.^54^ Besides the already mentioned verified risk factors of MAFLD, the search for other potential causes continues. Several studies proposed that periodontitis can negatively affect MAFLD onset and progression,^1,6,30^ while others attributed the MAFLD-periodontitis association to mutual risk factors such as obesity and metabolic syndrome.^76^

Obesity (BMI >35 kg/m^2^) is related to low-grade chronic systemic inflammation, which is accountable for the onset or aggravation of many obesity-related diseases, including MAFLD^71^ and periodontitis.^22,28,38^ Different pathways have been proposed as fostering periodontitis in obesity, including oxidative stress, proinflammatory cytokines (tumour necrosis factor-alpha),^22^ proinflammatory adipocytokines (leptin and resistin),^23^ monocytic myeloid-derived suppressor cells,^38^ alterations in innate and adaptive immune response, C-reactive protein (CRP),^22^ shared gene polymorphisms,^22^ as well as a shift in the intestinal and oral microbiome due to obesity.^41^ Caloric restriction is shown to lower both systemic and periodontal inflammation.^53^

On the other hand, periodontitis is a recognised risk factor for many systemic diseases marked by low-grade systemic inflammation, such as cardiovascular diseases,^14,56^ diabetes mellitus,^548,56^ neurological diseases,^27^ premature births,^13^ rheumatoid arthritis,^8^ and presumably MAFLD.^6,50^ It has been suggested that periodontitis-associated inflammatory mediators, bacteria, and their by-products (endotoxins) may seep into circulation and reach distant tissues, including the small intestine and liver.^30,59,60,77^ Oral bacteria associated with periodontal diseases can be found in the small intestine, potentially affecting the gut microbiome and liver tissue.^1,24,77^ Small intestine bacteria overgrowth (SIBO), marked by an increase in low-grade systemic inflammation, can promote the onset of MAFLD.^10^ Furthermore, studies have shown that periodontitis may increase the production of acute-phase proteins, such as CRP, independent of obesity or other causes.^57,72^ CRP is a non-specific biomarker of inflammation produced mainly in the liver and other cells, such as endothelial cells and lymphocytes.^66^ CRP produced locally and systemically in response to periodontitis might promote systemic inflammation and other insulin-resistance pathways that aggravate MAFLD.^6,41^ In accordance, a recent review reported a connection between periodontitis and MAFLD in insulin resistance and concomitant metabolic parameters.^76^ Therefore, the MAFLD-periodontitis association may ensue from shared risk factors, such as obesity, metabolic syndrome, and diabetes mellitus,^17,44,61,62^ as proposed by a recent review.^76^

Liver biopsy is still the gold standard in diagnosing the true extent of the MAFLD.^65^ Nevertheless, the majority of studies exploring the oral-liver relationship based the diagnosis of liver condition on non-invasive tests, i.e. ultrasonography or serum liver biomarkers, including gamma-glutamyl transferase (GGT).^6,30,32,34,76^ GGT is synthesised mainly in the liver and can be found in large quantities in the serum when the liver is damaged, but is also considered a non-specific marker of systemic inflammation and oxidative stress.^39^ In the available research ascertaining the MAFLD-periodontitis association, only patients with apparent signs of MAFLD were correctly diagnosed via liver biopsy.^7,49,77^ In contrast, most patients without apparent signs of MAFLD usually did not undergo a biopsy, therefore overlooking the actual presence of the liver pathology.^7,77^ In addition, the majority of the studies did not perform extensive dental and periodontal examinations^31,34^ or did not perform an oral clinical examination at all.^36,49,77^ Moreover, most studies failed to diagnose chronic gingivitis and included gingivitis patients among periodontally healthy individuals.^31,34^ It should be emphasised that previous studies on the periodontitis-MAFLD association failed to report data on obesity parameters.^4,7,64^ Consequently, further research on the periodontal-MAFLD association utilising robust diagnostic criteria and including all confounding factors is needed to determine the true nature of the MAFLD-periodontitis association.

Bariatric surgery is an effective treatment for morbid obesity and obesity-associated complications, and provides easier access to perform a liver biopsy.^16^ Hence, our study aimed to assess the prevalence of liver and periodontal pathology in a cohort of obese patients with BMI BMI > 40 undergoing bariatric surgery procedures and compare liver biopsy results and serum markers of liver disease between patients with and without periodontitis.

## Materials and Methods

### Study Design

The cross-sectional study included patients with obesity undergoing bariatric surgery. During the surgery, liver biopsy was performed and sent to pathophysiological analysis to determine the presence of MAFLD by Kleiner’s classification.^35^ Before surgery, all patients underwent detailed dental and periodontal examinations supported by radiographic findings to define their periodontal diagnosis according to the AAP/EFP classification.^73^ In addition, corresponding medical specialists examined bariatric surgery patients to diagnose/exclude obesity-related diseases. The following serum biochemical laboratory analyses were performed before surgery: serum levels of CRP, aspartate transaminase (AST), alanine transaminase (ALT), GGT, albumin, triglyceride, high-density lipoprotein (HDL), low-density lipoprotein (LDL) cholesterol, glucose and glycated haemoglobin (HBA1c).^26,65^ In addition, relevant demographic and behavioural data were collected from the medical records or interviews.

The study was conducted in accordance with the World Medical Association Declaration of Helsinki. Ethical approval for the study was received from Slovenia’s National Medical Ethics Committee (0120-312202010 and 0120-77/2019). All patients received a verbal and written explanation, then signed consent forms. In this cross-sectional study, the ‘Strengthening the Reporting of Observational Studies in Epidemiology’ (STROBE) guidelines were followed in conducting and presenting the research findings.^75.^

### Study Participants

The study included patients with obesity from April 2019 until December 2021 who underwent bariatric surgery and liver biopsy at the Department of Abdominal Surgery, University Medical Centre, Ljubljana, Slovenia. Liver biopsies were harvested during the bariatric surgery by the operating surgeon (TP). The 1-cm^2^ tissue biopsies were taken from the edges of liver segment III using an electrocautery instrument. The liver biopsies were placed in 10% formaldehyde and sent to the Institute for Pathology, Medical Faculty, University in Ljubljana, Slovenia, for histopathological evaluation.

Inclusion criteria were obese patients aged 18–70 years and indicated for bariatric surgery (BMI ≥40 kg/m^2^). Exclusion criteria were unwillingness to participate in the study; less than 15 teeth present; ASA III or IV or patients with other severe systemic diseases unrelated to obesity or liver disease; pregnant or lactating women; psychosis or other psychopathologies.

### Liver Pathology

Liver biopsy samples were prepared for pathohistological evaluation by the method described previously, i.e. biopsy specimens were stained with haematoxylin and eosin, reticulin and Masson trichrome stains.^12^ Kleiner’s classification was used to determine MAFLD presence and progression in the liver samples.^35^ The presence and degree of steatosis (0-3), hepatocyte ballooning (0-2), and lobular inflammation (0-3) were noted, together with the presence of liver fibrosis (0-4), by a single experienced pathologist.^35^ We classified pathophysiological changes of liver tissue based on Kleiner’s classification to determine the NAS activity score (stages 0-8) by combining the scores for steatosis, hepatocyte ballooning and inflammation. We used the NAS score to establish the diagnosis of non-NASH (NAS 0-2), borderline NASH (NAS 3,4) or NASH (NAS 5-8).^35^

### Dental and Periodontal Parameters

At the Department of Oral Medicine and Periodontology, all patients underwent a comprehensive dental and periodontal examination, with radiographic examinations performed by a single, experienced and calibrated examiner (RG) prior to bariatric surgery. The number of teeth, as well as fixed and removable partial dentures were evaluated during the dental and periodontal examination. The following periodontal parameters were recorded with the periodontal probe on each existing tooth, excluding third molars, at 6 sites: full-mouth plaque index (FMPI),^2^ probing pocket depth in (PPD) in mm, gingival recession (REC) in mm, bleeding on probing (BOP) (+/-), tooth mobility (stage 0–3),^48^ and furcation involvement (stage 0–3).^29^ Clinical attachment loss (CAL) was calculated post-hoc from PPD and REC. In addition, detailed radiographic analyses were done. If certain non-periodontal reasons for periodontal tissue destruction were suspected (e.g. iatrogenic cause, orthodontic anomalies, impacted 3rd molar distal to the 2nd molar, gingival recession of traumatic origin, and dental caries in the cervical area), such sites were excluded.

Periodontal diagnosis was made according to the AAP/EFP classification.^73^ To be categorised as a periodontitis case, patients had to exhibit detectable ≥ 1 mm interdental CAL or buccal/oral CAL of 3 mm with PPD of >3 mm on two or more non-adjacent teeth. Based on the clinical and radiological evaluation, periodontitis patients were further categorised by stages (I–IV) and grading system (A, B, C). Gingivitis in patients was diagnosed if there were ≥10% sites with BOP, with PPD ≤3 mm, and without CAL or bone loss.^15^ Patients were divided according to their periodontal diagnosis into a periodontitis group (PG) and a non-periodontitis group (NPG) (periodontally healthy patients and gingivitis patients).

### Systemic Health, Demographic and Behaviour Data

Demographic (sex, age, level of education) and anthropometric data (weight, height, waist size) were obtained from the medical records. As a part of the routine preoperative preparation, patients were checked for common obesity-related diseases: cardiovascular diseases, hypertension, endocrine diseases, diabetes mellitus, psychological disorders, obstructive sleep apnoea, dyslipidaemia (hypertriglyceridemia and/or hypercholesteremia) and presence of tmetabolic syndrome. The Edmonton Obesity Staging System (EOSS) score was determined.^63^ In the interview with the patient, information on behavioural habits, such as smoking (no/less/more than 10 cigarettes per day), drinking alcohol (more/less than 12 alcohol units a month), weekly exercise (more/less than 3 times a week for at least 20 min), daily oral hygiene (use of a toothbrush, toothpaste and interdental hygiene tools), and regular dental check-ups (at least twice a year), were collected.

### Sample Size and Statistical Analysis

Descriptive statistics and simple statistical tests (t-test for numerical values and Fisher’s exact test for categorical values) were used to evaluate the characteristics of the sample. α = 0.05 was set for all statistical tests. All statistical tests were performed with Microsoft Excel (Microsoft; Redmond, WA, USA).

## Results

The data presented in this study are available on request from the corresponding author.

### Sample Characteristics

The sample included 30 patients. Most of the patients were women (83%), mean age was 48.3 (SD 8.5), mean BMI was 43.5 kg/m^2^ (SD 8.2), while less than half of participants had higher education (40%). Around three-quarters of the included patients had metabolic syndrome (70%, 95% CI: 51–85%), dyslipidaemia (70%, 95% CI: 51–85%) and joint discomfort (77%, 95% CI: 58–90%), and more than 50% of patients had hypertension (67%, 95% CI:47–83%) and diabetes (60%, 95% CI:41–77%). Frequencies of obesity-related parameters and behavioural habits are presented in Table 1.

**Table 1:** Demographic and anthropometric data, systemic health, and behavioural characteristics of the sample (n = 30)

Parameters	Mean (SD); 95% CI or prevalence (%); 95% CI (n = 30)
Age (years)	48.3 (8.5); 30–66.9
Sex (females)	83%; 65–94%
Education (higher)	40%; 41–77%
BMI (kg/m^2^)	43.5 (8.2); 27.3–59.8
Waist (cm)	129.6 (15.1); 72–187
EOSS	2.5 (0.7); 1.6–3.5
Regular physical activity	34%; 18–54%
Smoking	34%; 18–54%
Alcohol consumption	10%; 2–27%
Obesity related diseases and conditions	
Metabolic syndrome	70%; 51–85%
Diabetes	60%; 41–77%
Hypertension	67%; 47–83%
Dyslipidaemia	70%; 51–85%
Sleep apnoea	34%; 18–54%
Joint discomfort	76%; 56–90%
Depression	21%; 8–40%
PCOS (females)	20%; 7–41%

CI: confidence interval; BMI: body mass index; EOSS: Edmonton Obesity Staging System; PCOS: polycystic ovary syndrome.

### Oral and Periodontal Health

The mean number of missing teeth among the examined patients was 8.3 (SD=6) for PG and 2.9 (SD=2.8) for NPG, while 21% of PG and none of the NPG wore removable dentures. The clinical and radiographic evaluations showed the prevalence of periodontitis was high at 66.7% (95% CI: 47–83%; 20/30), while the other patients manifested gingivitis (33.3%; 95% CI: 17–53%; 10/30 Table 2). There were no periodontally healthy patients in the sample. Periodontitis patients were further divided according to staging and grading of periodontitis, with around half the patients presenting with advanced stages (stage III and IV) and rapid progression rate (grade C) of periodontitis (Table 2). Periodontitis patients (n = 20) were included in PG, while gingivitis patients (n = 10) were included in NPG. The PG and NPG differed in all periodontal parameters, except for BOP. The number of missing teeth was significantly higher in the PG (p = 0.0046). In addition, the groups were similar in the number of crowns or dentures, dental visits, and adequacy of performing dental hygiene (Table 3).

**Table 2:** Periodontal diagnosis

				
Periodontal status (n = 30)	Periodontitis	Gingivitis		
	66.7%; 47–83% (20)	33.3%; 17–53% (10)		
Periodontitis stage (n = 20)	Stage I	Stage II	Stage III	Stage IV
	30%; 12–54% (6)	25%; 9–49% (5)	20%; 6–44% (4)	25%; 9–49% (5)
Periodontitis grade (n = 20)	Grade A	Grade B	Grade C	
	35%; 15–59% (7)	20%; 6–44% (4)	45%; 23–68% (9)	

All data are shown as prevalence, 95% confidence interval (number of patients).

**Table 3:** Oral and periodontal status of the sample and periodontitis and non-periodontitis (gingivitis) patients

Periodontal and oral parameters	Periodontitis (n = 20);	Gingivitis (n = 10);	Periodontitis vs gingivitis
BOP (%)	34.5 (24.5)	26.1 (13.5)	0.24 †
FMPI (%)	49.2 (27.1)	26.8 (16.1)	0.0097 † **
PPD (mm)	2.9 (0.5)	2.4 (0.2)	0.0035 † **
REC (mm)	1.4 (0.8)	0.8 (0.6)	0.0476 † *
CAL (mm)	1.5 (1.3)	0.2 (0.1)	0.0004 † ***
Tooth missing (no)	8.3 (6)	2.9 (2.8)	0.0046 † **
Tooth mobility (%)	6 (11.7)	0 (0)	0.043 † *
Crowns (no)	3.2 (4)	3.7 (6.7)	0.836 †
Denture (% of patients)	21%	0%	0.268 §
Regular dental visits (% of patients)	58%	80%	0.413 §
Adequate oral hygiene (% of patients)	37%	30%	1 §

All data are represented as mean (standard deviation) if not specified otherwise; PPD: probing pocket depth; REC: gingival recession; BOP: bleeding on probing; CAL: clinical attachment loss; FMPI: full-mouth plaque index; *p < 0.05; **p < 0.01; ***p < 0.001; †: t-test; §: Fisher’s exact test.

### Liver Health in the Sample

Pathohistological analysis of liver biopsy samples revealed a NASH (NAS 5–8) prevalence of 43% (95% CI: 25–63%), borderline NASH (NAS 3–4) 38% (95% CI:13–47%), and non-NASH (NAS 0–2) of 20% (95% CI: 8–39%). The most prevalent fibrosis stage was stage I, at 72% (95% CI: 54–88%; Table 4). The biochemical analysis of liver biomarkers in the serum is shown in Table 5. More than 50% of included patients had elevated CRP, LDL and lower HDL, and almost 50% had elevated ALT, triglycerides and glucose.

**Table 4:** Liver biopsy findings in the sample

Liver parameters (n = 30)				
NAS (0–8)	Non-NASH (NAS 0–2)	Borderline NASH (NAS 3–4)	NASH (NAS 5–8)	
	20%; 8–39% (6)	37%; 20–56% (11)	41%; 25–63% (13)	
Liver fibrosis (0–4)	0	1	2	3, 4
	13.3%; 4–31% (4)	73.3%; 54–88% (22)	13.3%; 4–31% (4)	0% (0)

NAS: non-alcoholic fatty liver disease activity score; NASH: non-alcoholic steatohepatitis; data shown as prevalence in the sample, 95% confidence interval (number of patients); P: number of periodontitis patients; G: number of gingivitis patients.

**Table 5:** Parameters of liver health in the sample, periodontitis, and gingivitis patients

Pathohistological liver biopsy evaluation	Sample (n = 30)	Periodontitis (n = 20)			Gingivitis (n = 10)			Periodontitis vs gingivitis; p-value
NAS (0-8)	4 (1.9)	3.7 (1.8)			4.8 (1.9)			0.107 †
NASH diagnosis		NASH	b-NASH	Non-NASH	NASH	b-NASH	Non-NASH	0.41 §
		7	8	5	6	3	1	
Liver fibrosis score		0	1	2	0	1	2	0.33 §
		2	14	4	2	8	0	
Serum biomarkers		Sample (n = 30)		Periodontitis (n = 20)		Gingivitis		Periodontitis vs gingivitis; p-value
Mean CRP (mg/l)		9.4 (6)		10.84 (6.20)		6.60 (3.84)		0.031 † *
CRP elevated (>5 mg/l)		59% (39–76%)		68% (43–87%)		40% (12–70%)		0.23 §
Mean AST (µkat/l)		0.44 (0.3)		0.45 (0.32)		0.43 (0.28)		0.89 †
AST elevated (>0.52 µkat/l)		18% (6–34%)		16% (3–35%)		22% (3–53%)		1 §
Mean ALT (µkat/l)		0.61 (0.26)		0.58 (0.20)		0.66 (0.35)		0.49 †
ALT elevated (>0.57 µkat/l)		43% (24–61%)		42% (20–64%)		44% (14–76%)		1 §
Mean Gama-glutamyl transferase (GGT)		0.48 (0.33)		0.36 (0.20)		0.73 (0.45)		0.03 † *
GGT elevated (>0.63 µkat/l)		21% (8–38%)		5% (0–19%)		56% (21–84%)		0.007 § **
Mean HDL (mmol/l)		1.25 (0.58)		1.31 (0.65)		1.14 (0.44)		0.25 †
HDL lowered (<1.3 mmol/l)		58% (37–77%)		47% (21–71%)		78% (40–97%)		0.21 §
Mean LDL (mmol/l)		3.43 (1.7)		3.26 (1.63)		3.72 (1.78)		0.41 †
LDL elevated (>3.4 mmol/l)		56% (34–74%)		56% (30–79%)		56% (21–84%)		1 §
Mean Triglyceride (mmol/l)		2.31 (2.84)		2.49 (3.41)		2.02 (1.37)		0.66 †
Triglyceride elevated (>1.7 mmol/l)		44% (25–63%)		35% (14–59%)		60% (26–86%)		0.25 §
Mean Albumin (g/L)		46 (12)		45.88 (14.85)		46 (3.59)		1 †
Albumin lowered (<32 g/L)		0%		0%		0%		1 §
Mean Glucose (mmol/l)		6.44 (2.52)		6.47 (2.94)		6.39 (1.53)		0.92 †
Glucose elevated (>5.6 mmol/l)		46% (28–65%)		39% (17–62%)		60% (26–86%)		0.43 §
Mean HBA1c (%)		4.84% (3.03)		4.48 (2.87)		5.21 (2.9)		0.57 †
HBA1c elevated (>6%)		31% (11–55%)		25% (3–58%)		38% (9–71%)		1 §

NAS: non–alcoholic fatty liver disease activity score; NASH: non–alcoholic steatohepatitis (NAS 5–8); b–NASH: borderline non–alcoholic steatohepatitis (NAS 3–4); non–NASH: non–non–alcoholic steatohepatitis (NAS 0–2); CRP: C reactive protein; AST: aspartate transaminase; ALT: alanine transaminase; GGT: gamma–glutamyl transpeptidase; HDL: high–density lipoprotein; LDL: low–density lipoprotein; HBA1c: glycated haemoglobin; all data presented as mean (standard deviation) or % of patients (95% confidence interval); †t–test; §Fisher’s exact test. *p < 0.05; **p < 0.01.

### Comparison of Liver and Systemic Parameters in Periodontitis vs Non-Periodontitis Patients

There were no differences between the PG and NPG in terms of systemic health, demographic or behavioural data. Among PG, most patients presented with borderline NASH (8/20), while more than 50% of NPG patients presented with NASH (6/10). There was no difference in NASH prevalence (p = 0.41), NAS (p = 0.107), or liver fibrosis (p = 0.33) between PG and NPG. Periodontitis patients presented higher CRP levels, while NPG patients had higher GGT (p < 0.05) (Table 5).

## Discussion

The present study showed a substantial prevalence of periodontitis, gingivitis and MAFLD among morbidly obese patients (BMI >40 ) undergoing for bariatric surgery. All periodontitis-resistant patients (NPG) were diagnosed with gingivitis, while no periodontally healthy patients were found. Among gingivitis patients, unexpectedly high levels of liver pathology were found, similar to periodontitis patients. Therefore, in addition to periodontitis-MAFLD, the gingivitis-MAFLD relationship should be explored in future studies. Furthermore, mild forms of liver fibrosis in the sample were high, but they did not correlate with periodontal diagnosis.

To the best of our knowledge, this is the first study in which morbidly obese patients have undergone liver biopsy and proper periodontal diagnostics. Even though we were unable to ascertain the correlation between periodontitis and the degree of MAFLD or liver fibrosis, this does not lessen the importance of examining periodontal conditions among obese patients scheduled for bariatric surgery. The prevalence of periodontitis in the sample was high, as expected for an obese cohort.^43,55^ Studies have shown that obesity negatively affects periodontal health, although the exact mechanism of interaction is still under investigation.^22^ Obesity-induced chronic systemic inflammation and dysfunction of immune response are the main reasons for periodontitis initiation and progression.^22,28^ However, other factors common in obesity, such as an unbalanced diet,^51^ eating disorders,^45,52^ comorbidities (e.g. diabetes mellitus,^58^ gastroesophageal reflux disease^67^) or medications (calcium channel blockers^20^) may also be involved.^22^ Furthermore, due to obesity, periodontal pathogen numbers are increased.^22,42^ Some data even suggest that obesity undermines the results of non-surgical periodontal therapy.^23^

As expected, in our study, periodontitis patients demonstrated higher CRP levels than did the non-periodontitis group.^3,32,67,80^ CRP, a marker of systemic inflammation, can also arise from obesity-driven low-grade systemic inflammation.^19,57^ However, previous research showed that periodontitis could increase CRP levels in obese patients by about 1 mg/l,^40^ whereas successful periodontal therapy could reduce CRP levels by about 1 mg/l CRP.^5,70,80^ On the other hand, higher GGT levels in the gingivitis group may reflect severe hepatocyte damage. Serum GGT is a measure of systemic oxidative stress that probably arises from MAFLD, obesity and other obesity-related diseases, manifesting as gingival inflammation.^9,18^ Therefore, we hypothesise that gingivitis in patients with MAFLD may result from liver pathology and associated systemic oxidative stress.^4^ GGT has been previously linked to periodontitis and bone metabolism,^47,68^ but not gingivitis.

In addition, changes in gut microbiota associated with MAFLD may shift the normal microbiota of the oral cavity,^1,25^ thereby initiating the inflammatory processes in the gingiva.^69^ Interestingly, some studies demonstrated a correlation between liver pathology and the quantity *P. gingivalis* in saliva,^77^ its presence in hepatocytes^21^ or serum antibodies against *P. gingivalis*.^49^ Furthermore, according to the few prospective studies, the history of periodontitis may be a risk factor for MAFLD, and vice-versa, the onset of MAFLD might negatively affect periodontal health.^4,37^ One way to indirectly substantiate the periodontitis-liver connection is with interventional studies. The effect of periodontal therapy on the liver was tested in a promising study,^33^ and previous research has already shown improvement in liver biomarkers after successful periodontal therapy.^77,80^ Similarly, periodontal therapy improved oral and gut microbiome in liver cirrhosis patients, decreasing systemic inflammatory markers such as IL-1β; IL-6.^11^

The main limitation of our study was the modest sample size. The second limitation, unbalanced groups, in addition to the small sample size, precludes any associative interpretation regarding MALDF and periodontitis. The high prevalence of both MAFLD and periodontitis in our sample may be explained by the obesity. Nevertheless, despite a small sample size, our study still detected higher CRP levels in obese periodontitis patients with MAFLD and higher GGT levels in obese MAFLD patients with gingivitis, but failed to detect other possible differences in cofounding factors between periodontitis and non-periodontitis patients. Therefore, the extension of our study with a larger sample and adjustment for confounding factors is required in the future.

## Conclusion

The study results suggest the considerable presence of both MAFLD and periodontitis in morbidly obese patients. Although we failed to find statistically significant differences in the extent or severity of MAFLD between patients with periodontitis and gingivitis, the high frequency of periodontitis among patients with BMI >40 still suggests the potential periodontitis-MAFLD association. Furthermore, morbidly obese patients with periodontitis had higher CRP levels, while those with gingivitis presented higher GGT levels. Our results justify further testing on a larger sample to adjust for the confounding factors, including obesity and diabetes.
